# An approach to the utilisation of CO_2 _as impregnating agent in steam pretreatment of sugar cane bagasse and leaves for ethanol production

**DOI:** 10.1186/1754-6834-3-7

**Published:** 2010-04-12

**Authors:** Viridiana Ferreira-Leitão, Clarissa Cruz Perrone, Joice Rodrigues, Ana Paula Machado Franke, Stefano Macrelli, Guido Zacchi

**Affiliations:** 1 National Institute of Technology, MCT, Rio de Janeiro, Brazil; 2 Chemical Engineering, Department of Chemical Engineering Lund University, Lund, Sweden

## Abstract

**Background:**

The conditions for steam pretreatment of sugar cane bagasse and leaves were studied using CO_2 _as an impregnating agent. The following conditions were investigated: time (5 to 15 min) and temperature (190 to 220°C). The pretreatment was assessed in terms of glucose and xylose yields after enzymatic hydrolysis and inhibitor formation (furfural and hydroxymethylfurfural) in the pretreatment. Results from pretreatment using SO_2 _as impregnating agent was used as reference.

**Results:**

For sugar cane bagasse, the highest glucose yield (86.6% of theoretical) was obtained after pretreatment at 205°C for 15 min. For sugar cane leaves the highest glucose yield (97.2% of theoretical) was obtained after pretreatment at 220°C for 5 min. The reference pretreatment, using impregnation with SO_2 _and performed at 190°C for 5 min, resulted in an overall glucose yield of 79.7% and 91.9% for bagasse and leaves, respectively.

**Conclusions:**

Comparable pretreatment performance was obtained with CO_2 _as compared to when SO_2 _is used, although higher temperature and pressure were needed. The results are encouraging as some characteristics of CO_2 _are very attractive, such as high availability, low cost, low toxicity, low corrosivity and low occupational risk.

## Background

Approximately 46% of the domestic supply of energy in Brazil is based on renewable energy, with 31% associated with the utilisation of biomass; 15.9% of the energy based on biomass is directly associated with sugar cane and its derivatives [1]. These figures are quite high when compared with those from the world energy matrix, where only 12.9% is associated with renewable energy. An even smaller ratio (6.7%) is observed when examining Organisation for Economic Co-operation and Development (OECD) countries [1].

Although Brazilian first-generation ethanol derived from sugar cane's sucrose is the only globally competitive biofuel (US$ 35 per barrel) [2], the production of second-generation ethanol from sugar lignocelluloses (for example, cane bagasse and leaves) is still a technological challenge. The utilisation of bagasse presents the additional advantage of being already available at the factory after processing for the extraction of the sugar cane juice. In the case of leaves, the Brazilian government is becoming more restrictive regarding the burning of residue in the field [3] and the development of more efficient burners [4-6] will also allow more efficient use of this resource. Even though both materials can be used in cogeneration of electricity, the increase of their value chain in a complementary way is certainly a welcome idea [7]. The utilisation of lignocellulosics hydrolysates as diluting agents in the molasses fermentation step could also overcome the problem of the low glucose concentration associated with these hydrolysates [8]. Additionally, Brazil is a country with an agricultural vocation and has a huge variety of agroindustrial residues, justifying the development of technologies for second-generation ethanol [9].

According to the above-mentioned reasons and considering that the raw material pretreatment step represents about 20% of the total costs of cellulosic ethanol production [10], the present study was focused on steam pretreatment of sugar cane bagasse and leaves using CO_2 _as an impregnating agent. The use of CO_2 _was investigated in order to explore some advantages of the gas: high availability in first-generation ethanol plants, low toxicity, low corrosivity and low occupational risk [11]. The efficiency of CO_2 _steam pretreatment was compared to the very efficient and commonly used SO_2 _steam pretreatment [12]. In the present study, steam pretreatment of CO_2 _impregnated sugar cane bagasse and leaves was investigated under conditions in the following ranges: time (5 to 15 min) and temperature (190 to 220°C). The pretreatment was assessed in terms of glucose and xylose yields after enzymatic hydrolysis and inhibitor formation (furfural and hydroxymethylfurfural) in the pretreatment. The results were also compared with pretreatment of sugar cane bagasse and leaves using SO_2 _and with pretreatment of leaves without any impregnation.

## Methods

### Raw materials

Fresh bagasse was kindly provided by Dr Jaime Finguerut (Centro de Tecnologia Canavieira, São Paulo, Brazil) and transported to Lund University, Sweden, by air. Sugar cane leaves were kindly provided by Dedini (São Paulo, Brazil) and also sent to Sweden by air. Both materials were assayed by the National Renewable Energy Laboratory (NREL) methods [13] for raw material composition (Table 1) and stored at 5°C. Sugar cane bagasse was used as received. The sugar cane leaves were washed three times with water after milling (approximately 2 × 10 mm size pieces) or cutting (approximately 2 × 10 cm size pieces) in order to remove the sand and debris. Sugar cane bagasse and leaves contained 67.1% and 89.0% of dry matter, respectively.

**Table 1:** Composition of sugar cane bagasse and leaves as percentage of dry matter

Content	Bagasse	Leaves
Glucan	41.4	33.3

Xylan	22.5	18.1

Arabinan	1.3	3.1

Galactan	1.3	1.5

Mannan	3.4	1.5

Lignin	23.6	36.1

Total	93.5	93.6

### Pretreatment

The pretreatment was performed in a 10 l steam pretreatment reactor as previously described [14]. Steam was provided using a 110 kW electrical boiler (Pann-Partner, Stockholm, Sweden). In the experiments with gas catalyst impregnation, the raw material was placed in a plastic bag and carbon or sulfur dioxide was supplied from a gas cylinder at atmospheric pressure. The amount of CO_2 _or SO_2 _added to the bag corresponded to 3% by weight based on the water content of the raw material (30% of dry matter), and was determined by weighing the bag before and after the addition of the gas [12]. After 2 h at room temperature (SO_2_) or 12 h at 5°C (CO_2_), the impregnated material was steam pretreated.

Bagasse and leaves corresponding to 225 and 560 g dry matter (DM), respectively, were loaded into the preheated 10 l reactor. Steam was supplied from a boiler and the material was heated in temperatures ranging from 190 to 220°C during 5, 10 or 15 min periods. Then, the pretreated material was discharged into a cyclone connected to the outlet of the reactor. The pretreated material was collected and samples from the supernatant were analysed to determine the yield of sugars and byproducts. When a high level of oligomeric sugar was observed in the supernatant, a subsequent step of acid hydrolysis was performed to convert all of it to its monomeric form. According to NREL procedures for total sugar analysis, both oligomers and monomers were determined in a 4% acid hydrolysis step [15].

The slurry samples withdrawn for analysis were washed thoroughly with water to remove soluble substances, dried at 105°C overnight and the content of water insoluble solids determined gravimetrically. The washed solids were also analysed by NREL methods [13] for carbohydrate, ash and lignin composition.

The pretreated sugar cane bagasse was separated through pressing to separate the solids from the liquid fraction. The solids were then washed thoroughly (50 ml/g of dry pretreated bagasse) with hot (approximately 50°C) water for 1 h under mechanical stirring and pressed again to remove the excess of washing water before the enzymatic hydrolysis assay. These solids were denoted water-insoluble solids (WIS). Pretreated sugar cane leaves were used without the separation process described above.

### Enzymatic hydrolysis

Enzymatic hydrolysis was carried out on the pretreated bagasse or leaves to evaluate the efficiency of the pretreatment using Celluclast 1.5 l (65 FPU/g and 17 β-glucosidase IU/g enzyme solution) and the β-glucosidase preparation Novozym 188 (376 β-glucosidase IU/g enzyme solution), kindly provided by Novozymes A/S (Bagsvaerd, Denmark). Enzymatic hydrolysis was performed in 1 l flasks at 40°C for 96 h using 2% (w/w) of pretreated material under mechanical stirring at 180 rpm. Only bagasse was previously washed before enzymatic hydrolysis.

The fibrous pretreated material (WIS sugar cane bagasse or non-washed sugar cane leaves) was placed in 1 l glass flasks, diluted with 0.1 M sodium acetate buffer (pH = 4.8) and mixed with Celluclast 1.5 l (15 FPU/g of fibrous material) and Novozym 188 (18 UI/g fibrous material). Buffer was added to a final weight of 600 g. All enzymatic hydrolysis trials were performed in two parallel runs and the average of the two runs was used.

### Analysis

The samples from pretreatment and enzymatic hydrolysis were analysed by high performance liquid chromatography (HPLC). All samples were filtered through a 0.20 μm filter and diluted prior to HPLC analysis. The concentration of cellobiose, glucose, xylose, galactose, mannose and arabinose in the liquid collected after pretreatment and in the samples from enzymatic hydrolysis were determined using an HPLC system (Shimadzu LC-10AD, Tokyo, Japan) equipped with a Refractive Index detector (Shimadzu RID-6A) and an Aminex HPX-87P column (Bio-Rad, Hercules, CA, USA) operating at 85°C with degassed ultrapure water (Millipore, Billerica, USA) as the mobile phase at a flow rate of 0.5 ml/min.

Furfural and hydroxymethylfurfural (HMF) in pretreatment liquid samples were analysed with an HPLC system (Waters, Milford, MA, USA) equipped with a Biosil C18 column (Bio-Rad) and detected using a UV detector (Waters 2487 dual absorbance detector) set on a wavelength of 220 nm and operating at room temperature. The mobile phase consisted of 40% (v/v) aqueous methanol, adjusted to pH 3 with concentrated HCl and supplied at a flow rate of 0.6 ml/min.

## Results and Discussion

The overall yields of glucose and xylose included the soluble sugars in the liquid from pretreatment and the soluble sugars obtained in the enzymatic hydrolysis. These overall sugar yields are expressed based on the percentage of the theoretical sugar content available in the raw material. Xylose yields in the liquid collected after pretreatment included both oligomeric and monomeric sugar. The results are a first screening of the possibility for use of CO_2 _as impregnating agent for pretreatment of sugar cane bagasse and leaves. The data measured are not sufficient for closing the mass balance as the amount of washed solids after pretreatment was not measured.

Table 1 shows the composition of the sugar cane bagasse and the leaves used in the present study. The sugar cane bagasse consists of 69.9% carbohydrates and 23.6% lignin, while the leaves consist of 57.5% carbohydrates and 36.1% lignin based on dry matter. The sugar cane bagasse has a higher content regarding of glucan and xylan than the leaves.

### Pretreatment and enzymatic hydrolysis

**Figure 1:**
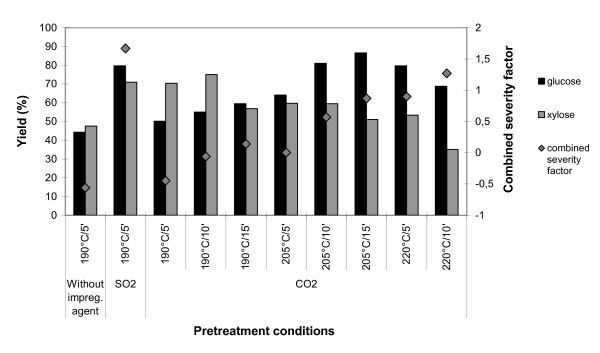
**Overall yields of glucose and xylose released in the pretreatment plus in the enzymatic hydrolysis of steam pretreated sugar cane bagasse at different conditions**. The pretreatment combined severity factor for each set is also shown.

According to Sendelius, the best condition for steam pretreatment of SO_2_-impregnated sugar cane bagasse, regarding sugar yield, was 190°C for 5 min, yielding 86.3% of glucose and 72.0% of xylose [12]. This condition was used for pretreatment of the bagasse in the present study and resulted in a pretreated material with an overall glucose yield of 79.7% after enzymatic hydrolysis. This pretreatment was used for comparison in the evaluation of CO_2_-impregnated bagasse steam pretreatment. Pretreatment of CO_2 _impregnated bagasse under the same conditions resulted in only a 50.2% glucose yield, as CO_2 _is a weaker acid impregnating agent. The final pH after pretreatment of bagasse at 190°C for 5 min without impregnating agent, with CO_2 _and with SO_2_, was 3.91, 3.80 and 1.68, respectively. Better results were obtained with the increase of temperature and time in the steam pretreatment (see Figure 1). Overall glucose yields of 81.1 and 86.6% were obtained after the enzymatic hydrolysis of steam pretreated CO_2_-impregnated bagasse performed at 205°C for 10 and 15 min, respectively.

**Figure 2:**
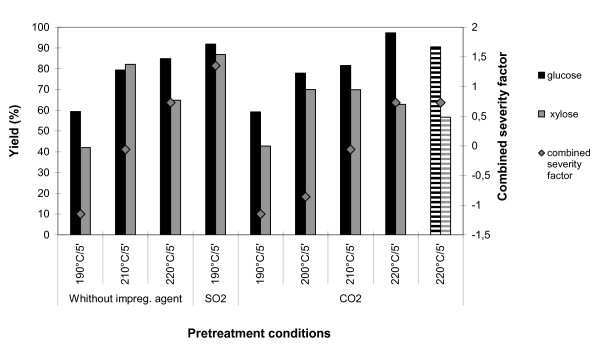
**Overall yields of glucose and xylose released in the pretreatment plus in the enzymatic hydrolysis of steam-pretreated sugar cane leaves at different conditions**. The pretreatment combined severity factor for each set is also shown. Striped columns correspond to cut, not milled, sugar cane leaves.

As no similar studies employing sugar cane leaves were found in the literature, the same set of conditions (190°C/5 min) were those initially tested to evaluate the susceptibility of sugar cane leaves against both impregnating agents (CO_2 _and SO_2_) and compared with the conditions already established for the bagasse. Figure 2 shows glucose yields of 59.4% (without impregnating agent), 59.3% (CO_2 _impregnation) and 91.9% (SO_2 _impregnation) under the previous conditions, establishing that the best impregnating agent for sugar cane leaves, at 190°C, is also SO_2_. The final pH after pretreatment of sugar cane leaves at 190°C for 5 min without impregnating agent, with CO_2 _and with SO_2_, was 4.5, 4.5 and 2.0, respectively. These results indicate that leaves have more buffering components than bagasse. The utilisation of higher temperatures in the steam pretreatment of CO_2_-impregnated leaves, however, turned out to generate pretreated material that furnished a very high glucose yield, 97.2%, after enzymatic hydrolysis of leaves pretreated at 220°C for 5 min. It is important to emphasise that the combined severity factor [16] indicated in all figures takes into account not only time and temperature of the steam pretreatment, but also the acidity generated in the reaction media by the addition of acid catalyst and the release of organic acids from the raw material, as indicated by pH measurement after pretreatment. The combined severity factor has been developed by Chum *et al*. [16] to account for the effect of introducing an acid as a pretreatment catalyst. The combined severity factor is calculated based on the severity factor R_0 _[17], which accounts for the effect of the steam temperature and the residence time at the steam temperature, and the pH after pretreatment, through the expression Log R_0_-pH, where Log R_0 _is given by the following equation:

Where t is time in minutes and T is the experimental temperature in °C.

**Figure 3:**
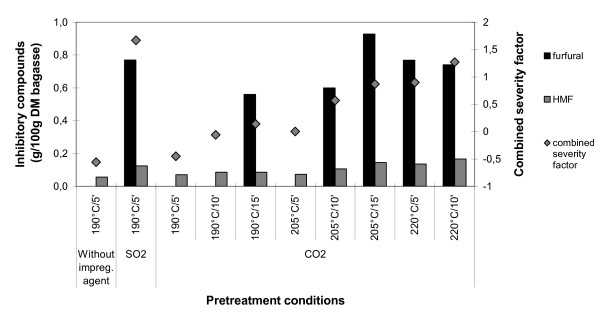
**Concentration of inhibitory byproducts in the hydrolysate obtained after the steam pretreatment of sugar cane bagasse at different conditions**. The pretreatment combined severity factor for each set is also shown. Furfural was not quantified for some of the samples due to problems with overlapping peaks in the high performance liquid chromatography (HPLC) chromatograms.

**Figure 4:**
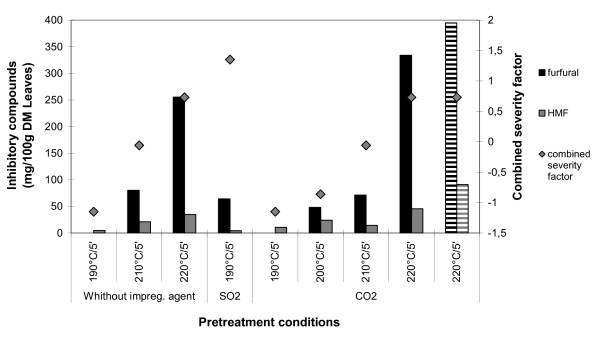
**Concentration of inhibitory byproducts in the hydrolysate obtained after the steam pretreatment of sugar cane leaves at different conditions**. The pretreatment combined severity factor for each set is also shown. Striped columns correspond to cut, not milled, sugar cane leaves. For two of the samples the concentration of furfural was low and could not be evaluated in the high performance liquid chromatography (HPLC) chromatogram due to overlap with other peaks.

The concentration of inhibitors after pretreatment is shown in Figures 3 and 4. There is a direct relationship between their concentration and the increase of temperature and/or time. However, it is also evident that impregnation with CO_2 _resulted in less degradation of the sugars released in the pretreatment compared to impregnation with sulfur dioxide. However, the formation of approximately similar amounts of furfural and HMF from sugar cane bagasse or leaves with CO_2 _as from the same material without impregnation implies that CO_2 _did not increase the degradation of sugars compared to the plain steam pretreatment. It should be clarified that furfural was presumably produced under all tested conditions but was not possible to quantify for some of the samples due to problems with overlapping peaks in the chromatograms. The concentration of acetic acid after pretreatment was determined only in the experiments carried out with the sugar cane leaves. The values obtained ranged from 0.24 to 1.03 g/100 g DM of leaves, respectively at 190°C and 220°C. There was no influence of the impregnating agent and a direct relationship was observed between the temperature increase and the acetic acid concentration. Although the effect of SO_2 _on the hydrolysis of the sugar cane bagasse and leaves steam pretreated at 190°C is superior, the utilisation of CO_2 _as impregnating agent allows the utilisation of higher temperatures and/or times in the pretreatment with only a small increase in sugars degradation. Although these conditions result in higher equipment costs, there are several advantages in the use of CO_2 _such as high availability, low cost, low toxicity, low corrosivity and low occupational risk.

Figure 1 shows an overall glucose yield of 79.7% for both SO_2_-impregnated bagasse pretreated at 190°C for 5 min and CO_2_-impregnated bagasse pretreated at 220°C for 5 min. A glucose yield of 86.6% was obtained for CO_2_-impregnated bagasse pretreated at 205°C for 15 min. These data and the corresponding chromatograms, shown in Figure 3, indicate that the utilisation of CO_2 _as impregnating agent at temperatures as high as 220°C and long residence times (15 min) provides enhancement of enzymatic hydrolysis of pretreated bagasse somewhat higher than those obtained using SO_2 _at 190°C without increasing the sugar degradation levels. As sugar release and inhibitor formation are two important factors for the efficiency of pretreatment, these preliminary findings are important in the evaluation of CO_2 _as an impregnating agent for steam pretreatment of sugar cane bagasse.

In the case of sugar cane leaves, the highest glucose yield (97.2%) for pretreatment after CO_2 _impregnation was obtained for pretreatment at 220°C for 5 min (Figure 2). However, under these conditions a more pronounced formation of inhibitors was achieved than for SO_2_-impregnated leaves pretreated at 190°C for 5 min (Figure 4). Despite the more pronounced formation of inhibitors, data from the literature show that these levels of furfural and hydroxymethylfurfural have no detrimental effects on the subsequent fermentation step [14]. The concentration of inhibitors for the aforementioned conditions can be considered low when compared to other impregnating agents, used in sugar cane bagasse pretreatment, for example H_2_SO_4 _[14]. It is also important to highlight the yield of glucose (90.5%) obtained using cut, not milled, CO_2_-impregnated sugar cane leaves pretreated at 220°C for 5 min. This glucose yield is slightly lower than that obtained for the same pretreatment conditions using the fine-milled material (97.2%). This indicates that it is possible to avoid the milling step in the raw material preparation of sugar cane leaves. However it has to be further investigated by economical analysis to see if the lower yields do compensate for the lower milling costs.

### Xylose release and pulp composition

**Figure 5:**
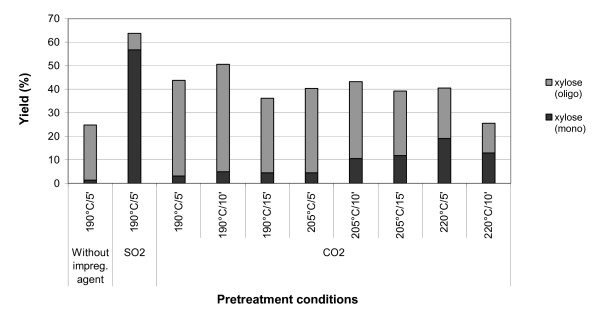
**Monomeric and oligomeric xylose yields in the hydrolysate obtained after the steam pretreatment of sugar cane bagasse at different conditions**.

**Figure 6:**
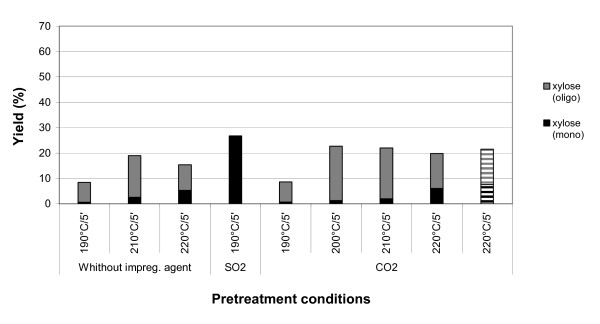
**Monomeric and oligomeric xylose (except for SO_2 _impregnation) yields in the hydrolysate obtained after the steam pretreatment of sugar cane leaves at different conditions**. Stripped columns correspond to cut, not milled, sugar cane leaves.

Figures 5 and 6 show the concentrations of xylose in monomer and oligomer forms (except for leaves impregnated with SO_2_) in the liquid fractions obtained after steam pretreatment (before enzymatic hydrolysis) of sugar cane bagasse and leaves, respectively, for the different sets of conditions employed. It is worth noting that the fraction of xylose that is released in oligomeric form in the liquid fraction was always higher than that released in monomer form for the tests without impregnation and for impregnation with CO_2_. The opposite behaviour is observed when SO_2 _is used as impregnating agent. This is obviously related to the higher acidity of H_2_SO_3 _compared to that of H_2_CO_3_, as previously mentioned. It is also important to emphasise that the content of xylose in the liquid fractions, before enzymatic hydrolysis, is always more abundant in bagasse than in leaves.

Tables 2 and 3 show the composition of the pulp after the pretreatment of bagasse and leaves before enzymatic hydrolysis. Confirming the previous results, xylan content in the pretreated leaf pulp is higher when compared to the percentage of xylan present in bagasse pulp.

**Table 2:** Composition of the solid fraction of pretreated sugar cane bagasse in percentage of dry matter before enzymatic hydrolysis and pH

Steam pretreatment conditions	Glucan	Xylan	Lignin	pH
190°C/5 min	46.9	13.0	26.8	3.9

190°C/5 min/SO_2_	49.7	3.2	29.2	1.7

190°C/5 min/CO_2_	43.5	10.6	26.3	3.8

190°C/10 min/CO_2_	52.0	8.2	27.6	3.7

190°C/15 min/CO_2_	49.8	12.0	27.6	3.7

205°C/5 min/CO_2_	53.0	9.5	27.2	3.8

205°C/10 min/CO_2_	60.5	6.5	29.5	3.5

205°C/15 min/CO_2_	59.3	4.5	30.8	3.4

220°C/5 min/CO_2_	59.1	4.0	31.2	3.3

220°C/10 min/CO_2_	58.0	3.0	32.6	3.2

**Table 3:** Composition of the solid fraction of pretreated sugar cane leaves in percentage of dry matter before enzymatic hydrolysis

Steam pretreatment conditions	Glucan	Xylan	Lignin	pH
190°C/5 min	39.2	19.7	35.0	4.5

210°C/5 min	40.7	7.4	33.8	4.0

220°C/5 min	53.7	2.2	37.9	3.5

190°C/5 min/SO_2_	52.3	8.7	33.6	2.0

190°C/5 min/CO_2_	39.2	19.2	30.0	4.5

200°C/5 min/CO_2_	43.6	16.7	29.2	4.5

210°C/5 min/CO_2_	50.9	9.7	35.0	4.0

220°C/5 min/CO_2_	52.7	4.2	41.5	3.5

220°C/5 min/CO_2 _(cut leaves)	53.8	2.9	37.8	3.5

## Conclusions

The utilisation of CO_2 _as an alternative impregnating agent for steam pretreatment is promising. Despite the low solubility of CO_2 _and the higher temperatures that are needed for providing pretreated materials with increased accessibility to enzymatic hydrolysis, some advantages in its utilisation are very attractive, such as high availability, low toxicity, low corrosivity and low occupational risk.

This study showed that sugar cane bagasse and leaves require different pretreatment conditions for steam pretreatment to obtain maximum sugar yields. Despite their differences, it was possible to find a set of pretreatment conditions that would allow the steam pretreatment of sugar cane bagasse and leaves together simultaneously (for example, CO_2 _impregnation and pretreatment at 220°C for 5 min). Although the present study comprised only the pretreatment of both materials separately, the aforementioned conditions provided interesting yields: 97.2% of glucose and 62.8% of xylose for the leaves and 79.7% of glucose and 53.3% of xylose for bagasse. Further experiments are required to evaluate the proportion of each material that should be used.

There are few studies using the leaves of sugar cane as a raw material for the production of ethanol. Despite the advantages offered by the bagasse, the leaves are abundant and may be potentially interesting for the production of this important biofuel. In this study it was shown that this material can be used milled or cut, with very promising yields. The flexibility in the use of bagasse and leaves, both for the production of alcohol and for cogeneration, also associated with the production of sugar and ethanol from sucrose, is an interesting strategy. Thus it is important to develop technology that allows for this flexibility of production according to market demands. Another relevant point is that residual lignin from second-generation ethanol could be also used for cogeneration.

## Competing interests

CP and GZ are the authors of a Brazilian patent (BR 0803354-4, reference number 11) that is being currently applied for by the National Institute of Technology. The patent describes a process for biomass steam pretreatment using CO2 as impregnating agent. The applicant was CP's employer during the execution of the experimental work.

## Authors' contributions

VL and CP designed and carried out the experiments with sugar cane leaves and bagasse, respectively, analysed the results and wrote the manuscript. JE participated in the general pretreatment work of leaves and analysed the composition of pretreated bagasse with AF. SM participated in the experimental designing of leaves pretreatment and helped draft the manuscript. GZ participated in the experimental designing and reviewed the manuscript. All authors read and approved the final manuscript.
